# Butyrate Produced by Gut Microbiota Regulates Atherosclerosis: A Narrative Review of the Latest Findings

**DOI:** 10.3390/ijms26146744

**Published:** 2025-07-14

**Authors:** Leon M. T. Dicks

**Affiliations:** Department of Microbiology, Stellenbosch University, Private Bag X1, Matieland, Stellenbosch 7602, South Africa; lmtd@sun.ac.za

**Keywords:** atherosclerosis, butyrate, gut microbiota

## Abstract

Atherosclerosis (AS), a progressive inflammatory disease of coronary arteries, the aorta, and the internal carotid artery, is considered one of the main contributors to cardiovascular disorders. Blood flow is restricted by accumulating lipid-rich macrophages (foam cells), calcium, fibrin, and cellular debris into plaques on the intima of arterial walls. Butyrate maintains gut barrier integrity and modulates immune responses. Butyrate regulates G-protein-coupled receptor (GPCR) signaling and activates nuclear factor kappa-B (NF-κB), activator protein-1 (AP-1), and interferon regulatory factors (IFRs) involved in the production of proinflammatory cytokines. Depending on the inflammatory stimuli, butyrate may also inactivate NF-κB, resulting in the suppression of proinflammatory cytokines and the stimulation of anti-inflammatory cytokines. Butyrate modulates mitogen-activated protein kinase (MAPK) to promote or suppress macrophage inflammation, muscle cell growth, apoptosis, and the uptake of oxidized low-density lipoprotein (ox-LDL) in macrophages. Activation of the peroxisome proliferator-activated receptor γ (PPARγ) pathway plays a role in lipid metabolism, inflammation, and cell differentiation. Butyrate inhibits interferon γ (IFN-γ) signaling and suppresses NOD-, LRR-, and pyrin domain-containing protein 3 (NLRP3) involved in inflammation and scar tissue formation. The dual role of butyrate in AS is discussed by addressing the interactions between butyrate, intestinal epithelial cells (IECs), endothelial cells (ECs) of the main arteries, and immune cells. Signals generated from these interactions may be applied in the diagnosis and intervention of AS. Reporters to detect early AS is suggested. This narrative review covers the most recent findings published in PubMed and Crossref databases.

## 1. Introduction

According to 2024 statistics, more than 20 million natural deaths in 2021 were ascribed to atherosclerosis (AS) and other cardiovascular diseases (CVDs) [1]. AS is defined as chronic inflammation and thickening of arteries, associated with the deposition of plaques formed from the accumulation of fatty acids, calcium, fibrin, and cellular debris. This leads to restricted blood flow and a deficiency in oxygen supply (hypoxia) to the heart, brain, kidneys, pelvis, and limbs [2,3,4]. Rupturing plaques leads to blood clot formation and thrombosis [5]. With the progression of AS, granulocytes, monocytes, macrophages, and dendritic cells accumulate at the site of infection, increasing the risk of plaque rupture, myocardial infarction (MI), and thrombosis [6]. Although diet and genetics play important roles in developing AS, external factors such as stress, smoking, abnormal alcohol consumption, lack of exercise, and diabetes are considered major risk factors [7,8,9,10,11,12].

Individuals following a Western-style diet with a high intake of processed foods, refined grains, red and processed meats, and high sugar content are more prone to developing AS and other CVDs [13,14,15]. The gut microbiome of these individuals is dominated by *Firmicutes* (e.g., *Streptococcus* and *Oscillibacter*), *Proteobacteria* (*Bilophila*), *Pseudomonadota* (e.g., *Enterobacterium*, *Escherichia coli*, and *Desulfovibrio*), and *Fusobacteriota* (*Fusobacterium*), and contains fewer *Bacteroidetes* (e.g., *Bacteroides*, *Parabacteroides Prevotella*, *Barnesiella*, and *Alistipes*), and *Actinobacteria* (e.g., *Bifidobacterium*, *Streptomyces*, and *Actinoplanes*) [16,17,18]. Individuals following a high-fiber plant-based Mediterranean diet (MD) are less prone to developing AS [19], which is attributed to a gut microbiome dominated by short-chain fatty acid (SCFA)-producing bacteria, such as members of the genera *Bifidobacterium*, *Lactobacillus*, *Enterococcus*, *Lachnospiraceae*, *Ruthenibacterium*, *Flavonifractor*, *Ruminococcus* [15,20,21,22], *Faecalibacterium*, *Roseburia*, *Eubacterium*, *Anaerostipes*, *Coprococcus*, *Subdoligranulum*, *Anaerobutyricum*, and *Oscillospira* [23,24,25,26,27]. Butyrate is mainly produced in the large intestinal tract [28,29,30]. Several studies have linked butyrate to the protection of CVDs [27,31], including the prevention of AS [32,33].

**Figure 1:**
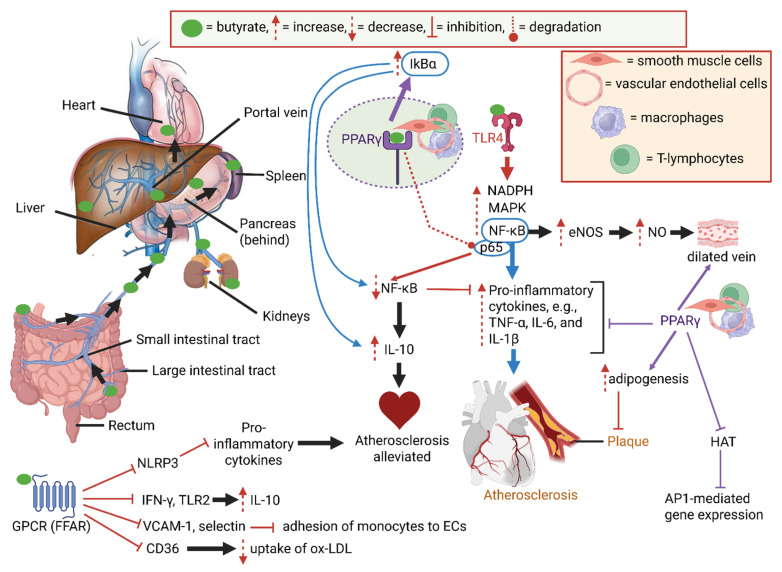
Butyrate produced by gut microbiota, mainly in the large intestine, enters the portal vein and is distributed to various organs (a few listed here). Interaction with Toll-like receptor 4 (TLR4) leads to an increase in nicotinamide adenine dinucleotide phosphate (NADPH), mitogen-activated protein kinase (MAPK), and nuclear factor kappa B (NF-κB). The latter activates the expression of endothelial nitric oxide synthase (eNOS), and nitric oxide (NO) levels increase, which acts as a vasodilator. NF-κB stimulates the formation of proinflammatory cytokines (e.g., TNF-α, IL-6, and IL-1β) and initiates plaque formation. The binding of butyrate to peroxisome proliferator-activated receptor γ (PPARγ) downregulates NF-κB production and upregulates the production of anti-inflammatory cytokines such as IL-10. The latter suppresses atherosclerotic activities. PPARγ suppresses the production of proinflammatory cytokines and the activity of histone acetylase (HAT). By suppressing HAT, DNA is not acetylated, and genes are not expressed. PPARγ also stimulates the activity of IkBα, an inhibitor of the NF-κB pathway, and promotes the production of anti-inflammatory cytokines such as IL-10. PPARγ also induces adipogenesis and prevents the accumulation of lipids on atrial walls and plaque formation (shown as a red, healthy heart). The binding of butyrate to G-protein-coupled receptors (GPCRs) suppresses the production of interferon γ (IFN-γ), TLR2, and Nod-like receptor pyrin domain 3 (NLRP3), and downregulates the production of adhesion molecules such as vascular cell adhesion molecule 1 (VCAM-1) and E-selectin—all suppressing the production of proinflammatory cytokines. The suppression of interferon γ (IFN-γ) and Toll-like receptor 2 (TLR2) by butyrate suppresses the activity of the glycoprotein CD36 and decreases the uptake of oxidized low-density lipoprotein (ox-LDL) by macrophages. ECs = endothelial cells, AP1 = activator protein-1, red heart symbol = a healthy heart (atherosclerosis alleviated). The interactions between compounds are shown by using different colored arrows. The dotted line between PPARγ and p65 refers to ligation. The schematic representation was constructed using Biorender (Biorender.com), accessed on the 18 June 2025.

Butyrate, produced by gut bacteria in the colon, diffuses across the gut wall or is actively transported by proton-coupled monocarboxylate transporters. Butyrate in the circulatory system is transported via the portal vein to various organs (Figure 1). Butyrate activates toll-like receptors (TLRs) and attaches to membrane-linked G-protein-coupled receptors (GPCRs), also referred to as free fatty acid receptors (FFARs), and peroxisome proliferator-activated receptors (PPARs) to regulate enzymatic and immunological pathways, as summarized in Figure 1 [27,34,35]. Activation of the TLR4 pathway by butyrate leads to an increase in nicotinamide adenine dinucleotide phosphate (NADPH), activation of the mitogen-activated protein kinase (MAPK) pathway, and an increase in nuclear factor kappa B (NF-κB) (Figure 1) [36]. An increase in NF-κB activates endothelial nitric oxide synthase (eNOS) expression, which increases nitric oxide (NO) production (Figure 1). This potent vasodilator may help to ease restricted blood flow caused by plaque formation [37,38,39]. Proinflammatory cytokines (e.g., TNF-α, IL-6, and IL-1β), produced from induced NF-κB, initiate the formation of plaques and atherosclerotic lesions [40] (Figure 1).

PPARγ, expressed in monocytes/macrophages, T-lymphocytes, vascular endothelial cells (VECs), and smooth muscle cells (SMCs), acts as an E3 ubiquitin ligase with p65 (Figure 1), degrading the NF-κB/p65 complex [41], resulting in the downregulation of the NF-κB pathway [42,43]. PPARγ also stimulates the activity of IkBα (Figure 1), an inhibitor of the NF-κB pathway [44], and promotes the production of anti-inflammatory cytokines such as IL-10, which helps to resolve atherosclerotic inflammation and stimulates the production of insulin [33,45]. Activated PPARγ suppresses the production of proinflammatory cytokines [42,46] and induces adipogenesis (the formation of fat cells) (Figure 1), thereby preventing the accumulation of lipids on atrial walls [47]. PPARγ also plays an important role in keeping arteries dilated and regulating blood pressure (Figure 1) [42]. PPARγ competes with activator protein 1 (AP1) for binding to coactivators such as p300 and the CREB-binding protein (CBP) that acetylates histones (Figure 1). This prevents AP1-mediated gene expression (Figure 1). Dysregulation of PPARγ leads to hypertension, obesity, high blood sugar, elevated serum triglycerides, and a decrease in high-density lipoprotein (HDL) [48]. Butyrate also suppresses the activity of the Nod-like receptor pyrin domain 3 (NLRP3) inflammasome (Figure 1), thereby preventing the release of proinflammatory cytokines and reducing plaque formation [49,50]. Butyrate may also exert anti-inflammatory activities by suppressing the production of interferon γ (IFN-γ) and Toll-like receptor 2 (TLR2) [51,52,53,54] and decreases the uptake of oxidized low-density lipoprotein (ox-LDL) in macrophages by suppressing the activity of CD36 (Figure 1), a glycoprotein with high affinity for lipids [33]. Du et al. [55] have shown that butyrate downregulates genes involved in lipid metabolism, including acyl-CoA thioesterase1 (Acot1), Acot2, Perilipin2 (Plin2), Plin5, and Cytochrome4a (10, 14, and 31 isoforms), and activates ATP-binding cassette subfamily A member 1 (ABCA1), leading to a reduction in total cholesterol (TC) and a decline in the accumulation of plaques. Butyrate also downregulates the overproduction of adhesion molecules such as vascular cell adhesion molecule 1 (VCAM-1) and E-selectin (Figure 1). By doing this, the adhesion of monocytes to injured endothelium is prevented or reduced [56].

Although most studies support the positive effect of butyrate in preventing or suppressing AS, a recent study [57] has shown that butyrate, orally administered (3.9 g of sodium butyrate daily for 4 weeks), increased daytime systolic and diastolic blood pressure in patients with hypertension. Although not directly linked to AS, an increase in blood pressure may cause damage to restricted arteries. The authors, however, acknowledged that contradictory and inconsistent results were reported with animal studies and that further research is required to understand the interaction between butyrate and its receptors pertaining to the regulation of blood pressure. The authors also acknowledged that their study has several limitations, e.g., only 23 patients were included in the trial, the nighttime blood pressure readings of patients who received butyrate did not vary significantly, and although the differences recorded with daytime readings were statistically significant, the variations were modest. These findings will have to be confirmed with extensive trials. A study conducted by Gee et al. [58] has shown that the activation of NF-κB may accelerate certain cardiovascular abnormalities. The findings from this study suggest that NF-κB induces the transformation of endothelial cells into mesenchymal cells in the aorta, leading to calcification and stenosis, which is characteristic of calcific aortic valve disease (CAVD). Although the study did not link NF-κB activation to butyrate, the induction of pathways involved in the production of proinflammatory cytokines (e.g., TNF-α) instigated an endothelial-to-mesenchymal transformation in aortic valve endothelial cells. As mentioned elsewhere, butyrate regulates the TLR4 pathway and activates NF-κB, which induces the production of proinflammatory cytokines, including TNF-α [40]. Reports such as these emphasize the complexity of AS and the role of butyrate in CVDs and CAVD. This necessitates an in-depth understanding of the interactions between butyrate, intestinal epithelial cells (IECs), endothelial cells (ECs), mesenchymal cells, and immune cells.

Butyrate has distinct effects on vascular smooth muscle cells (VSMCs) and systemic adipose tissue. In VSMCs, butyrate acts as an antiproliferative agent, inhibiting cell growth and migration, which is beneficial for preventing CVDs. In adipose tissue, butyrate reduces the accumulation of lipids and regulates energy consumption, potentially playing a role in obesity management and metabolic health. In this review, the preventative role of butyrate in AS, and its role in cardiovascular health are discussed. The first part of the review addresses the production of butyrate by gut microbiota and its uptake by intestinal epithelial cells (IECs). This is followed by a discussion of the interactions between butyrate and the endothelial cells (ECs) of arteries, its transfer across the endothelium, and its alteration of the immune system. The impact of signals generated from these interactions on cardiovascular health is discussed, with emphasis on AS. The possibility of using signals generated by the various interactions in the early diagnosis and intervention of AS is discussed. This narrative review covers the most recent findings, mostly from papers published in PubMed and Crossref databases.

## 2. Degradation of Carbohydrates and Production of Butyrate in the Large Intestinal Tract

Food-derived glycans, such as starch, sucrose, and lactose, are degraded in the small intestine of humans by at least 17 glycoside hydrolases [59]. The glycosidic bonds of amylose and amylopectin are hydrolyzed by α-amylases secreted from parotid, submandibular, and sublingual saliva glands [60]. In the small intestine, most of the salivary amylases are inactivated by gastric acid. The remaining polysaccharides are degraded by pancreatic amylases and enzyme complexes attached to the plasma membrane of mucosal cells [59]. Monomers are absorbed from the small intestinal lumen and either diffuse across the membrane of IECs or are actively transported with dedicated transport proteins. Complex (polymeric) carbohydrates in the diet (e.g., cellulose, hemicellulose, lignin, pectin, and mucilage and also mucins (glycoproteins with O-linked carbohydrates)), produced by goblet cells in the intestinal epithelial lining, need to be depolymerized before being transferred across IECs [61].

The human gastrointestinal tract (GIT) lacks the enzymes required to degrade complex indigestible carbohydrates [61] and depends on enzymes produced by gut microbiota. These enzymes include glycoside hydrolases, polysaccharide lyases, carbohydrate esterases, and glycosyltransferases [62]. *Akkermansia muciniphila* produces mucin-degrading glycoside hydrolases (GH16) that degrade mucin to acetate, which is then converted into butyrate by *Faecalibacterium prausnitzii* [63]. Most of the insoluble lipids (triglycerides) ingested are emulsified by bile salts, degraded by pancreatic lipases to energy-rich free fatty acids (FFAs) and monoacylglycerols, and then absorbed by enterocytes in the small intestine [64]. A small percentage of oxidized FFAs are re-esterified to triacylglycerols, absorbed at the apical side of enterocytes in the duodenum and proximal jejunum, transported to the endoplasmic reticulum (ER), and taken up by chylomicrons (large lipoprotein particles) before being secreted from the basolateral side into the circulatory system [65,66].

Lipids reaching the colon are mostly the short-chain fatty acids (SCFAs) butyrate, propionate, and acetate. Since most butyrate and other SCFAs in a diet are readily absorbed in the ileum, the levels that reach the colon are inadequate to support the cellular functions of enterocytes, including the synthesis of DNA, RNA, proteins, and lipids [30,67,68]. The 15 to 20% butyrate produced by colonic gut microbiota supplies approximately 70% of the energy to IECs [28,29,30]. Most of the butyrate and other SCFAs are produced from the fermentation of unfermented fructooligosaccharides (FOS), inulin, sugar alcohols, starch, cellulose, hemicellulose, lignin, pectin, and gum (mucilage) by *Lactobacillus*, *Bifodobacterium*, *Lachnospiraceae*, *Blautia*, *Coprococcus*, *Roseburia*, *Eubacterium*, *Faecalibacterium*, *Anaerostipes*, *Enterococcus*, *Phascolarctobacterium*, *Akkermansia*, *Ruminococcus*, and *Lachnospiraceae* [23,68,69,70]. Propionate, produced at similar levels, is rapidly absorbed in the rectosigmoid [71]. High levels of acetate (50 to 60%) are produced by colonic bacteria [29]. However, approximately 24% of the acetate is converted into butyrate [72,73], and the rest is absorbed by colonic enterocytes through non-ionic diffusion or by Na^+^-acetate co-transport [74,75]. Isobutyrate, a branched-chain fatty acid (BCFA), produced by gut microbiota from valine, may provide additional energy to IECs [76,77,78]. Although other BCFAs, such as 2-methyl butyrate and isovalerate, are produced from the fermentation of isoleucine and leucine, respectively, these are less absorbed by IECs [78,79]. BCFAs are predominantly produced by rumen bacteria and have, thus, not been studied in humans to the same extent as SCFAs [80]. BCFAs have, however, been detected in microbial biofilms that formed on the skin of fetuses, in colostrum, serum, and adipose tissue [80].

**Figure 2:**
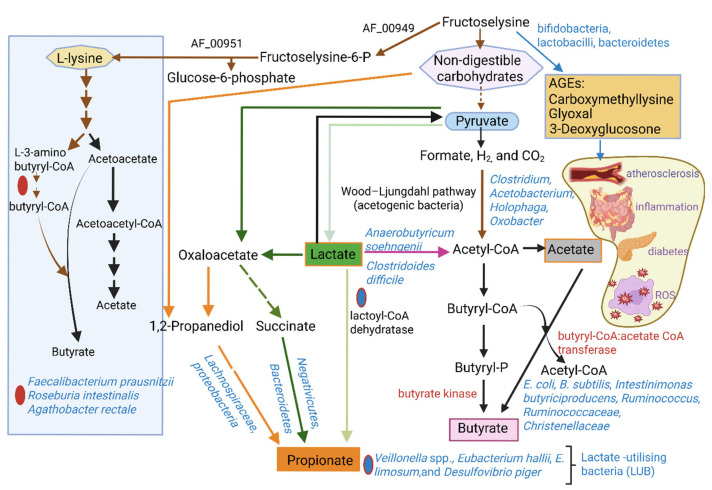
Pathways used by gut microbiota to metabolize non-digestible carbohydrates and amino acids (e.g., lysine) to butyrate and other short-chain fatty acids such as propionate and acetate. Fructoselysine (top of diagram) is phosphorylated by fructoselysine kinase (AF_00949) to form fructoselysine-6-phosphate, which is subsequently cleaved by fructoseamine deglycase (AF_00951) into lysine and glucose-6-phosphate (brown arrows). L-lysine is degraded via the lysine pathway to acetate and butyrate (blue box). This pathway is followed by *Faecalibacterium*, *Roseburia*, and *Agathobacter*. The production of lactate depends on the levels of pyruvate available and the redox state. In the presence of acetate (abundantly present in the colon), fructoselysine is converted into approximately three moles of butyrate (two moles of butyrate are formed via butyryl-CaA:acetate CoA transferase and butyrate kinase, and one mole of butyrate via the lysine pathway). Lactate is an intermediate that is either directly converted into propionate by lactoyl-CoA dehydratase (light green arrow) or indirectly via propanediol and succinate via oxaloacetate (dark green and orange arrows). Propanediol may also be produced via other routes from non-digestible carbohydrates. In the absence of exogenous acetate, fructoselysine is converted into approximately two moles of butyrate and one mol of lactate. Advanced glycation end products (AGEs), formed from fructoselysine (brown box), attach to proteins that may stimulate inflammation, increase the production of reactive oxygen species (ROS), and lead to plaque formation and diabetes (light yellow circle). For more detailed information on the pathways, the reader is referred to Ríos-Covián et al. [78] and Anshory et al. [95]. The red dot denotes the pathway used by *Faecalibacterium*, *Roseburia*, and *Agathobacter* spp, and the blue dot the pathway used by *Veillonella* spp., *Eubacterium hallii*, *Eubacterium limosum*, and *Desulfovibrio piger*. Each pathway is shown by using different colored arrows. The schematic representation was constructed using Biorender (Biorender.com), accessed on the 18 March 2025.

The production of butyrate by gut microbiota is summarized in Figure 2. Acetogenic anaerobic chemolithoautotrophic bacteria (e.g., *Clostridium*, *Acetobacterium*, *Holophaga*, and *Oxobacter*) convert formate, H_2_, and CO_2_ to acetate via the Wood–Ljungdahl pathway (brown arrows) [81,82,83]. Additional acetate is converted to butyrate by butyryl-CoA:acetate CoA transferase (encoded by *but*) or butyrate kinase (encoded by *buk*) (black lines) [84,85]. Butyrate may also be produced from the fermentation of fructoselysine and lysine via butyryl-CoA (brown arrows in blue box). Fructoselysine is a glycol-amino acid that forms at high temperatures in certain heat-processed foods [86]. *Flavonifractor plautii* converts L-lysine into crotonyl-CoA (not shown), which is then hydrogenated by *Faecalibacterium prausnitzii*, *Roseburia intestinalis*, and *Agathobacter rectale* into butyryl-CoA [85,87,88]. Most anaerobic bacteria degrade lysine via the pathways depicted in the blue box. Propionate is produced from the intermediate 1,2-propanediol, which is converted to propionaldehyde), and subsequently to propionate (orange arrows). This pathway is used by proteobacteria and members of the *Lachnospiraceae* family [82,89]. Propionate may also be formed via the succinate pathway, i.e., via the decarboxylation of methylmalonyl-CoA to propionyl-CoA and the deacetylation of succinyl-CoA (not shown) to succinate, which is then decarboxylated to propionate (dark green arrows). This pathway is used by *Firmicutes* belonging to the *Negativicutes* class, and *Bacteroidetes* [90]. Lactate is converted to propionate via the acrylate pathway, with lactoyl-CoA dehydratase as the key enzyme (light green arrows). This pathway is used by only a few bacteria classified as lactate-utilizing bacteria (LUB), e.g., *Veillonella* spp., *Eubacterium hallii*, *E. limosum*, and *Desulfovibrio piger* [91,92,93,94].

Fructoselysine is a key intermediate in the formation of advanced glycation end products (AGEs), such as carboxymethyllysine [96] and reactive α-dicarbonyls (i.e., glyoxal and 3-deoxyglucosone; blue line to brown box) [97]. AGEs attach to proteins and may lead to plaque buildup in arteries [98]. AGEs are also associated with chronic inflammation, diabetes, Crohn’s disease [99,100], and oxidative stress (listed in the light-yellow area) [101]. In a microbially balanced GIT, fructoselysine and AGEs are degraded to butyrate by *Escherichia coli*, *Bacillus subtilis*, *Intestinimonas butyriciproducens*, *Ruminococcus* spp., and members of the families *Ruminococcaceae* and *Christenellaceae* [85,86].

Gut microbiota unable to ferment complex carbohydrates obtain their energy through substrate cross-feeding, i.e., the fermentation of metabolites produced by hydrolytic bacteria. *Roseburia intestinalis*, *F. prausnitzii*, and *Agathobacter rectale* cross-feed on acetate (blue box) [102], *Anaerobutyricum soehngenii* on lactate [87], and *Clostridoides difficile* on succinate [103] to produce butyrate (green box and purple and black lines). *Bifidobacterium* spp. uses cross-feeding to produce SCFAs from inulin-type fructans (ITFs) and oligosaccharides released by inulin-degrading microbiota [104]. *Bacteroides uniformis* and *Escherichia coli* grown in co-culture degrade agaro-oligosaccharides (AOs) [105]. *Bifidobacterium adolescentis* and *Bifidobacterium infantis* ferment agarotriose, an intermediate from the degradation of AOs [105]. Another example of cross-feeding is butyrate production by the lactate-negative (unable to ferment lactate) *Roseburia* sp. strain A2-183 when co-cultured with *Bifidobacterium adolescentis* L2-32 [92].

## 3. Absorption and Uptake of Butyrate by Colonic Intestinal Epithelial Cells (IECs)

**Figure 3:**
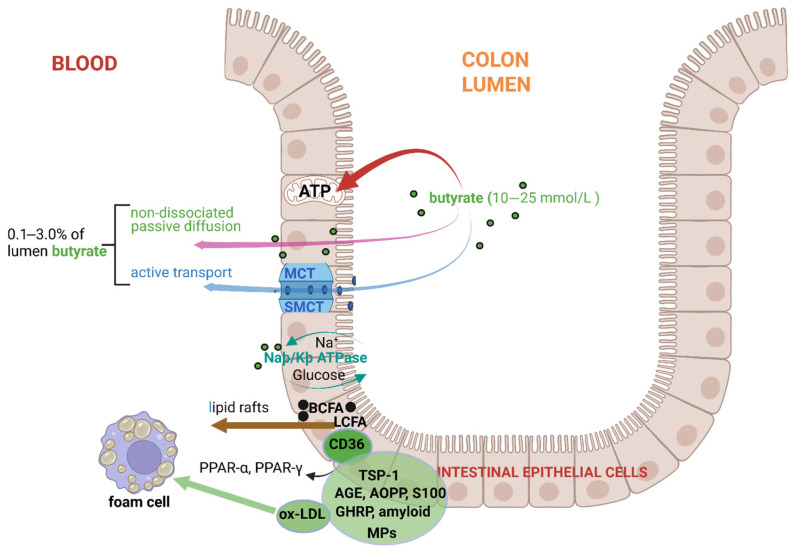
Butyrate, absorbed by colonocytes in the proximal colon, enters the TCA cycle to produce ATP (red arrow). Butyrate that crosses the gut wall, either by diffusion (pink arrow) or with the assistance of proton-coupled monocarboxylate transporters (MCTs) and sodium-coupled monocarboxylate transporters (SMCT), depicted in blue, enters the circulatory system. Butyrate is also transferred across the gut wall by transmembrane glycoprotein CD36 (cluster of differentiation 36), which has a high affinity for oxidized low-density lipoprotein (ox-LDL), shown in green circles. Ox-LDL is taken up by specific macrophages (foam cells). CD-36 also binds to proteins such as thrombospondin (e.g, TSP-1); advanced glycation end products (AGEs); advanced oxidation protein products (AOPPs); S100 family proteins S100-A8, S100-A9, and S100-A12 that bind Ca^2+^; growth hormone-releasing peptide (GHRP); cell-derived microparticles (MPs); and amyloids. Branched-chain fatty acids (BCFAs) and long-chain fatty acids (LCFAs) are transported across the plasma membrane via “lipid rafts” (brown arrow). The green dots denote butyrate, and the black dots BCFAs. The schematic representation was constructed using Biorender (Biorender.com), accessed on the 18 June 2025.

Most of the butyrate in the large intestinal tract is absorbed by colonocytes in the proximal colon [105,106] and transported to the mitochondria, where it is converted to acetyl-CoA in the TCA cycle to produce ATP (red arrow, Figure 3) [30]. Only 0.1% of the butyrate in the lumen of the large intestinal tract is transferred to the circulatory system [107]. However, experiments with radioactive butyrate have shown that this figure could be as high as 2.0–3.0% in healthy individuals [72]. Butyrate diffuses across the gut wall (pink arrow, Figure 3) or is transported across the apical membrane of colonocytes with the assistance of proton-coupled monocarboxylate-transporters [MCT1 (SLC16A1) or MCT4] and sodium-coupled monocarboxylate transporters [SMCT1 (SLC5A8) and SMCT2 (SLC5A12)] (blue arrow, Figure 3) [108,109,110]. At a pH below neutral, butyrate transported with MCTs is reduced (coupled to H^+^) and inhibited by acetate, propionate, pyruvate, lactate, and α-ketobutyrate [111]. The highest levels of MCT1 are found in the distal colon [112]. SMCT1, dependent on Na^+^, is more active in the ileum [113]. Once in the bloodstream, butyrate is transferred via the portal vein to the liver and converted to glucose via gluconeogenesis [114]. Branched-chain fatty acids (BCFAs) and long-chain fatty acids (LCFAs) are transported across the plasma membrane via “lipid rafts” (brown arrow, Figure 3) composed of glycosphingolipids, cholesterol, and special structured proteins [115]. Elevated levels of BCFAs in the liver may lead to non-alcoholic fatty liver disease (NAFLD) [116]. LCFAs are oxidized in the liver to triacylglycerols (TAGs) and converted to ketones that serve as a source of energy [117]. The accumulation of LCFAs, such as palmitic acid, can activate hepatic stellate cells and lead to liver fibrosis [117].

SCFAs, including butyrate, are also transferred across the gut wall by transmembrane glycoprotein CD36 (cluster of differentiation 36), known as fatty acid translocase glycoprotein IIIb (FAT GPIIIb), platelet glycoprotein IV (GPIV), 88 kD membrane protein (GP88), and scavenger receptor class B type 2 (SR-B2). Although CD36 has a high affinity for oxidized low-density lipoprotein (ox-LDL), LCFAs, and phospholipids, it also binds to proteins such as thrombospondin (e.g., TSP-1); advanced glycation end products (AGEs); advanced oxidation protein products (AOPPs); S100 family proteins S100-A8, S100-A9, and S100-A12 that bind Ca^2+^; growth-hormone-releasing peptide (GHRP); cell-derived microparticles (MPs); and amyloids (Figure 3) [118,119,120]. Despite being a double-transmembrane protein, CD36 does not form a channel through which fatty acids are transported [121]. The highly hydrophobic outer ring of CD36 serves as a docking site for fatty acids and other hydrophobic ligands [122]. For further information on the role of CD36 in the regulation of lipid homeostasis, the reader is referred to the review by Shu et al. [118].

## 4. Transport of Butyrate Across Arterial and Myocardial Endothelia

Although butyrate transport across colonic epithelial cells is well-studied, less is known about butyrate transport across arterial and myocardial endothelia [32,122]. Some evidence suggests that butyrate is transported across these endothelia using carrier proteins similar to those found in the colonic epithelium. MCTs may, thus, be used to transport butyrate across the epithelium [123,124]. The function of MCTs is regulated by a complex interplay of genetic, transcriptional, post-translational, and protein–protein interactions. An in-depth study of the transcription factors and signaling pathways that influence the expression of MCT genes may provide a better understanding of the conditions associated with butyrate transport across epithelia and the selection of reporter molecules to follow butyrate trafficking. Further information on MCT regulation may also lead to the treatment of AS, CVDS, and other diseases where MCTs play a role, e.g., cancer, diabetes, and neurological disorders. This is especially important in AS, as changes in the metabolism of macrophages, e.g., a shift from oxidative phosphorylation to glycolysis, may be an early sign of plaque formation. Genes and gene products orchestrating such a metabolic shift may provide clues to the development of diagnostic tests that predict changes in macrophage behavior. This will not only foresee plaque formation but also provide more information on the behavior, polarization, inflammatory responses, and stability of macrophages in atherosclerotic lesions.

Undissociated (non-ionized) butyrate may diffuse across the lipid-rich myocardial endothelial membrane. Fatty acids (FAs) and glucose, metabolized in the mitochondria of myocardial cells, supply most of the energy to the cardiovascular system [114], thus necessitating an active transport system, such as that driven by the glycoprotein CD36. This supplies 70% of the energy required by myocardial cells to sustain contractions [125]. Dysregulation in the synthesis of CD36 results in irregular myocardial contractions [126,127] and a decrease in PPAR-α, which results in the accumulation of toxic lipids and heart failure [128]. With the high affinity of CD36 to ox-LDL and LCFAs, it is not surprising that CD36 stimulates platelet formation [129]. CD36, oriented on the surface of foam cells, accumulates AGEs, accelerating the formation of atherosclerotic plaques [130] and diabetes [131,132]. ox-LDL stimulates PPAR-γ, the main transcription factor of CD36, thus leading to the acceleration in ox-LDL uptake and the formation of foam cells [133]. Because CD36 is involved in lipid accumulation, foam cell formation, inflammation, endothelial apoptosis, and thrombosis, it is the ideal basis from which a reporter system may be developed and used in the early detection of AS. Further research on the regulation of gene *CD36*, transcription factors such as PPAR, conditions leading to PPAR heterodimer formation, and PPAR response elements is required to develop a ligand that could be used as a CD36 reporter. This is a challenging task, as CD36 is a diverse protein that binds to LCFAs, oxLDL, oxidized phospholipids, thrombospondins, amyloid proteins, collagen, AGE, and anionic phospholipids.

## 5. Butyrate Interactions with G-Protein-Coupled Receptors (GPCRs)

GPCRs serve as docking stations for butyrate and other SCFAs, lipids, hormones, neurotransmitters, chemokines, sugars, proteins, Ca^2+^, guanosine triphosphate (GTP), and guanosine diphosphate (GDP) [134]. Class A GPCRs (the “rhodopsin-like family”) are the most diverse and represent several subgroups (aminergic, peptide, protein, lipid, melatonin, nucleotide, steroid, alicarboxylic acid, sensory, and orphan) [135,136]. Most of the class A GPCRs (75%) are aminergic. Approximately 10% are peptide ligand receptors, associated with analgesics, allergies, cardiovascular diseases, hypertension, pulmonary diseases, depression, migraine, glaucoma, Parkinson’s disease, schizophrenia, and cancers [137]. Six percent of class A GPCRs serve as docking stations for sensory molecules and alicarboxylic acids (e.g., SCFAs). Other GPCRs with relevance to humans are from class B (secretins), class C (metabotropic glutamates), and class F (“frizzled/smoothened”) [137].

Butyrate binds to G-protein-coupled receptors GPCR41 (free fatty acid receptor 3; FFAR3), GPCR43 (FFAR2), and GPCR109A (hydroxycarboxylic acid receptor 2, HCAR2) on the surface of IECs and ECs [138]. GPCR41 and GPCR43 are highly expressed in intestinal enteroendocrine cells (EECs) and regulate hormone and peptide production [139] but are also expressed in vascular smooth muscle cells (VSMCs) [140]. GPCR41 is strongly expressed by most cells [111,141], while GPCR43 is mainly expressed by lymphatic and immune cells [142,143]. GPCR41 has a strong affinity for butyrate and GPCR43 for acetate [142]. GPCR124, also known as tumor endothelial marker 5 (TEM5), plays a crucial role in the angiogenesis of the central nervous system (CNS) and development of the blood–brain barrier (BBB) [144]. GPCR124 also functions as a co-activator of protein WNT7, a signaling molecule involved in angiogenesis, the activation of nitrosative stress, and NLRP3 inflammasome signaling. The manipulation of endothelial GPCR124 may repress inflammatory responses and prevent AS. Manipulation of lysophosphatidic acid (LPA) GPCRs (LPARs) may repress the metabolism of lipids and platelet formation, thus preventing AS [145]. LPA enhances the expression of monocyte chemotactic protein-1 (MCP-1), which attracts monocytes and macrophages to sites of inflammation via the Gαi-RhoA-ROCK-NF-κB-dependent signaling pathway [146]. In-depth studies on LPA biosynthesis, metabolism, and signaling pathways may be a viable strategy for preventing and treating atherosclerosis and thrombosis.

**Figure 4:**
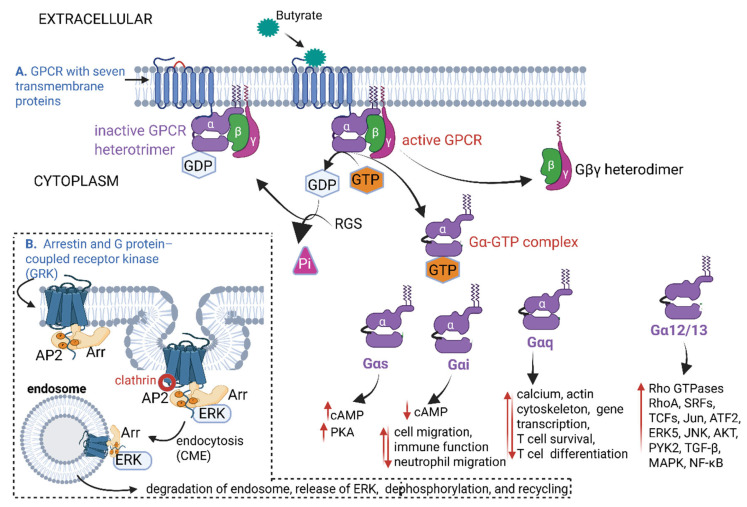
A schematic representation of activities stimulated and repressed when butyrate (blue-green dots) binds to G-protein-coupled receptors (GPCRs). (**A**) With the attachment of butyrate to the GPCR, the G-protein complex is activated. Upon dissociation of the Gα-protein from the Gα-GTP complex, several cellular reactions are stimulated (red arrow pointing upwards) and repressed (arrow pointing downwards). (**B**) Arrestin proteins (Arr) attach to extracellular signal-regulated serine/threonine kinase (ERK), adaptor protein 2 (AP2, orange circles), and clathrin (red circle) to form a clathrin-coated pit. The clathrin-coated pit splices off the plasma membrane and is taken up by a clathrin-coated vesicle (endosome). ERK released from degraded and dephosphorylated endosomes is recycled and either stimulates atherosclerogenic processes or inhibits atherosclerosis (AS) by removing apoptotic cells. RGS = regulator of G protein signaling; Gαs = Gα stimulatory protein; Gαi = Gα inhibitory protein; Gαq = Gα ubiquitous protein; Gα12/13 = Gα signaling protein; cAMP = cyclic adenosine monophosphate; PKA = cAMP-dependent protein kinase; Rho = transcription termination factor; SRF = serum response factor (a transcription factor); TCF = T-cell factor/lymphoid enhancer factor (a transcription factor); Jun = transcription factor complex, which is a key component of AP2 (Activator Protein-2); ATF2 = activating transcription factor 2; MAPK = mitogen-activated protein kinase; JNK = c-Jun N-terminal kinase; AKT or PKB = protein kinase B (a serine/threonine kinase); NF-κB = nuclear factor kappa B; TGF-β = transforming growth factor β. The schematic representation was constructed using Biorender (Biorender.com), accessed on the 18 June 2025.

With the binding of butyrate to GPCRs (Figure 4) and manipulating of the LPA receptor, the progression of AS may be repressed. GPCRs are embedded into cell membranes with seven α-helix transmembrane proteins (TM1 to TM7), as shown in Figure 4A. The TM proteins are linked by three intracellular loops (IL1–IL3) and three extracellular loops (EL1–EL3) (shown as black half circles on both sides of the TM proteins). A conserved disulfide bond between TM3 and TM4 stabilizes the orientation of the TM complex (red half circle, Figure 4A). TM7 is attached to three G-protein subunits (α, β, and γ). In the inactive form of GPCR, the Gα-protein is attached to GDP and remains strongly linked to the Gβγ heterodimer (Figure 4A). With the attachment of butyrate to the GPCR, GDP is replaced by GTP, and the G-protein complex is activated (Figure 4A). GTPase activity is controlled by regulatory G protein signals (RGS) and effector enzymes such as adenylyl cyclases (cyclic AMP and cAMP) [147]. This changes the conformation of the Gα-protein, and the Gα-GTP complex dissociates from the Gβγ subunit (Figure 4A). The Gα-proteins transform into four variations, i.e., stimulatory Gαs, inhibitory Gαi, ubiquitous Gαq, and signaling Gα12/13. Each variation in these Gα-proteins controls specific cellular functions and pathways (Figure 4A). Activated GPCRs also interact with arrestin proteins (Arr), as shown in Figure 4B, top part of the dotted line insert. Clathrin (red circle) binds to adaptor protein 2 (AP2, orange circles), attached to the Arr-extracellular signal-regulated serine/threonine kinase (ERK) complex to form a clathrin-coated pit. The clathrin-coated pit splices off from the plasma membrane (Figure 4B) and is taken up by the clathrin-coated vesicle (endosome). The ERK, released from degraded and dephosphorylated endosomes, is recycled (Figure 4B) and dysregulates inflammatory processes, leading to the activation of atherosclerogenic reactions [148]. However, ERK may also remove apoptotic cells and inhibit AS [149]. ERK is an anti-inflammatory signal that suppresses expression of NF-κB-dependent inflammatory genes by inhibiting IκB kinase activity in endothelial cells [149]. Thus far, four Arr proteins have been identified, e.g., Arr-1 (visual arrestin), Arr-2 (β-arrestin-1), Arr-3 (β-arrestin-2), and Arr-4 (cone arrestin) [150]. Each Arr protein has a conserved domain that binds to a specific GPCR. Phosphorylation of the receptor C-terminal tail by a GPCR kinase (GRK) promotes Arr recruitment and activation, including endocytosis through interactions with clathrin [151].

Class A GPCRs have a higher preference for β-arrestin-2 over β-arrestin-1, whereas Class B GPCRs do not display a preference [152]. Class B GPCRs interact with β-arrestins that are attracted to the angiotensin II type 1A receptor (AT1aR), the muscarinic acetylcholine receptor (mAChR), and the parathyroid hormone 1 receptor (PTH1R). Although both β-arrestins have an affinity for these receptors, their interactions may have different functions [153] and mAChRs are internalized without the preference of a specific β-arrestin [154]. Both β-arrestin-1 and β-arrestin-2 are recruited to the active PTH1R, but β-arrestin-1 seems to stabilize the PTH1R conformation and promotes receptor internalization via more phosphorylation sites than β-arrestin-2 [155].

The roles of β-arrestin-1 and β-arrestin-2 in the regulation of smooth muscle cell proliferation and migration, inflammation, and autophagy in macrophages suggest that they have a complex role in AS. Recent studies have shown that β-arrestin-1 induces the proliferation of endothelial cells and restores endothelial functions, including tube formation. Ma et al. [156], Alamanda et al. [157], and Shao et al. [158] have shown a dramatic increase in macrophage foam cells and plasma and cellular cholesterol levels in mice mutants that could not produce β-arrestin-1. However, an increase in the expression of β-arrestin-1 by macrophages led to a decrease in foam cell formation and an increase in autophagy, which resulted in the alleviation of AS [158]. β-arrestin-1 acts as a scaffold or adaptor protein involved in several GPCR-dependent or independent signaling pathways [158]. The search for ligands binding to GPCR may lead to the development of novel methods to detect early signs of AS. Studies on radioactive and fluorogenic ligands need to be pursued. Research should also focus on the dysregulation of β-arrestin as a method to control AS.

## 6. The Role of Nuclear Factor Kappa-B (NF-κB) in Atherosclerosis

The binding of butyrate to GPCRs activates the Gαi-PLC-PKC-ERK1/2 and the Gαq-PLC-IP3-[Ca^2+^]i pathways [159], regulating Ca^2+^ gradients, actin formation, and the differentiation of T cells. Butyrate also activates the TLR4 pathway, leading to an increase in the production of NADPH, activation of the MAPK pathway, and an increase in the expression of NF-κB, which, in turn, enhances the activity of eNOS and increases NO levels, as discussed elsewhere in this review. The overproduction of NF-κB induces the expression of proinflammatory cytokines, e.g., tumor necrosis factor-alpha (TNF-α), interferon-gamma (IFN-γ), IL-1β, IL-6, IL-8, IL-17, IL-12, and anti-inflammatory cytokines IL-10 and transforming growth factor beta (TGF-β) [160,161,162]. Activation of NF-κB also increases the expression of genes such as hydroxymethylglutaryl (HMG)-CoA reductase (HMGCR), which is involved in cholesterol biosynthesis, and the LDL receptor (LDLR) required for the uptake of LDL into cells [55,163]. NF-κB is also activated by fibroblast growth factors (FGFs), particularly FGF1 and FGF2, which induce the phosphorylation of protein p65 (also known as RelA), a key transcription factor of the NF-κB complex [164,165]. Furthermore, NF-κB initiates the production of Twist (a helix-loop-helix protein), the transcription factors Snai1 and Snai2 (also known as Slug), and cytoplasmic nuclear-factor-activated T cells (NFATc1) [166], which alters the polarity of ECs and enhances their transition to mesenchymal cells (referred to as epithelial–mesenchymal transition, EMT), contributing to the initiation of AS [167]. Normally, protein p65 is degraded by chaperone-mediated autophagy (CMA), resulting in the downregulation of NF-κB [164]. However, with the onset of EMT, CMA is suppressed, leading to a further increase in NF-κB activity [164]. Mesenchymal cells are more permeable than ECs and, thus, more susceptible to leukocyte trafficking, which is characteristic of inflamed and angiogenic cells [166]. In some instances, EMT is reversible, i.e., mesenchymal cells may be restored to ECs when inflammatory and angiogenic stimuli subside. However, in pathogenic settings such as AS, the transformation into mesenchymal cells is permanent [167]. In mesenchymal cells, the production of adherin junction molecules such as vascular endothelial (VE)–cadherin, is downregulated, whereas the production of α-smooth muscle actin (α-SMA) and collagen deposition is upregulated [167]. Mesenchymal cells are fibrotic, thicker, and stiffer than normal ECs due to changes in the cytoskeleton and an increase in extracellular matrix (ECM) deposition [167]. Plaques form when monocytes, macrophages, foam cells, plasma proteins, collagen (I and II), elastin, and cell debris attach to the ECM proteins [168].

With the activation of NF-κB, the levels of GTP cyclohydrolase (GTPCH) decrease, resulting in a decline in the production of tetrahydrobiopterin (BH4 or THB) [169] and the production of NO instead of reactive oxygen species (ROS), such as O_2_^−^ [170]. NO inhibits the expression of monocyte chemoattractant protein-1 (MCP-1), thus preventing the recruitment of monocytes [171]. NO also increases the expression of prostacyclin (prostaglandin) in damaged ECs [172], which relaxes VSMCs (reduces blood pressure) and prevents plaque formation [173,174]. Thus, by lowering the levels of GTPCH or suppressing its activity, the risk of hypertension and plaque formation is reduced [49,175]. However, an increase in GTPCH and BH4 activity induces the uncoupling of eNOS, leading to the production of cell-damaging ROS and AS [176,177,178,179]. Increased production of O_2_^−^ is normally observed in damaged and aged vessels [178]. Damaged cells may produce higher levels of protein kinases, e.g., serine/threonine kinase Akt (protein kinase B, also known as Rac kinase), which phosphorylates eNOS and increases its activity and, thus, NO production [180,181,182]. Peroxynitrite (ONOO^−^), formed from the reaction between O_2_^−^ and NO, binds to phospholipase A2 and inhibits the release of arachidonic acid [183,184]. A decrease in arachidonic acid results in lesser production of cyclooxygenase-2 (COX-2) and lower levels of prostacyclin, which may lead to hypertension and plaque formation [185,186]. The levels of ONOO^−^ production are, thus, controlled by BH4 [187,188]. At high oxidative stress, BH4 is converted by dihydrofolate reductase to 7,8-dihydrobiopterin (BH2). The latter facilitates the uncoupling of eNOS, thus preventing the conversion of L-arginine to NO [188]. A decline in NO production negatively affects the expression of vascular cell adhesion molecule-1 (VCAM-1), intercellular adhesion molecule-1 (ICAM-1), and E-selectin, all important adhesion molecules involved in the recruitment of leukocytes [189,190].

Butyrate acts as a negative regulator of the phosphatidylinositol 3-kinase (PI3K) or protein kinase B (Akt) signaling (PI3K/Akt) pathway and prevents the phosphorylation and activation of Akt. Inactivation of the PI3K/Akt pathway leads to reduced cell growth, increased inflammation, and irregular contractions of heart muscles. This causes ventricular arrhythmia (abnormal and irregular heartbeat that starts in the lower chambers of the heart), atrial fibrillation (abnormal heart rhythm), sinus node disease that affects the heart’s natural pacemaker (sinus node) and, thus, rhythm, and, if not treated, cardiac death [191].

## 7. Activation of Aryl Hydrocarbon Receptor (AhR) Proteins

Aryl hydrocarbon receptor (AhR) proteins are abundantly expressed in the endothelium and VSMCs [192,193,194]. AhR proteins were originally described as hepatic intracellular proteins with high affinity to the carcinogen 2,3,7,8-tetrachlorodibenzo-p-dioxin (TCDD) [195]. Subsequent studies have shown that AhR and AhR-ligands play a major role in cardio physiology [196,197], myocardial injury [198], AS [199], vascular development, and blood pressure [200,201,202].

**Figure 5:**
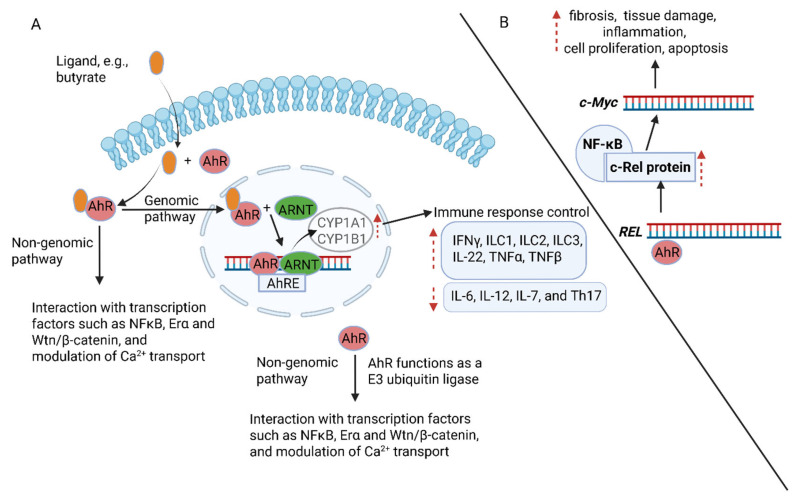
Transfer of butyrate into the nucleus. (**A**) Butyrate (orange dot) is transferred into the nucleus with the assistance of an aryl hydrocarbon receptor (AhR). The butyrate-AhR complex binds to the aryl hydrocarbon receptor nuclear translocator (ARNT) and the AhR element (AhRE). The butyrate-induced AhR-ARNT-AhRE complex promotes the transcription of several genes encoding the production of cytokines, cytochrome P450 family 1 subfamily A member 1 (CYP1A1), and CYP1B1. AhR also suppresses the production of certain cytokines, thus acting as an immune modulator. (**B**) The binding of AhR to the proto-oncogene *REL* encodes the c-Rel protein, a subunit of NF-κB. This activates the transcription of the c-Myc gene that drives fibrosis, inflammation, proliferation, apoptosis, and certain cancers. AhR also modulates immune responses, inflammation, and calcium transport across cell membranes. Dotted arrows refer to activation (showing upwards) and repression (showing downwards). The schematic representation was constructed using Biorender (Biorender.com), accessed on the 18 May 2025.

AhRs attach to multiple molecules, e.g., SCFAs (including butyrate); indole-3-acetic acid and indole-3-aldehydes produced from the degradation of tryptophan (Trp); halogenated aromatic hydrocarbons (HAHs); polycyclic aromatic hydrocarbons (PAHs), such as pyrenes, 2,3,7,8-tetrachlorodibenzo-p-dioxin (TCDD); polyunsaturated fatty acids such as arachidonic acid (AA); and flavonoids (pyrene linked to two benzene rings) [203,204,205,206]. In the genomic signaling pathway, butyrate activates AhR and transfers the butyrate-AhR complex to the nucleus, where it binds to the aryl hydrocarbon receptor nuclear translocator (ARNT) and the AhR element (AhRE) (Figure 5A). The butyrate-induced AhR-ARNT-AhRE complex promotes the transcription of several genes, e.g., cytochrome P450 family 1 subfamily A member 1 (CYP1A1), CYP1B1, and cytokines IL-1, IFNγ, IL-C1, IL-C2, IL-C3, IL-22, TNFα, and TNFβ (Figure 5A) [199]. AhR also suppresses the production of cytokines, such as IL-6, IL-12, IL-7, and Th17 (Figure 5A) [195]. In the non-genomic pathway (Figure 5A), AhR functions as an E3 ubiquitin ligase. The released c-SRC kinase phosphorylates multiple targets, e.g., regulating protein degradation. These non-genomic pathways modulate immune responses, inflammation, and calcium transport across cell membranes (Figure 5A) [207,208]. The binding of AhR to the proto-oncogene *REL* encodes the c-Rel protein, a subunit of NF-κB that activates the transcription of the c-Myc gene (Figure 5B). The latter drives organ fibrosis in tissue remodeling or tissue damage and regulates inflammation, proliferation, apoptosis, and certain cancers [209,210]. A homolog of c-Rel, v-rel avian reticuloendotheliosis viral oncogene homolog A (RELA), found in the avian retrovirus Rev-T, regulates the expression of AhR in VSMCs [211], stimulating their development [212]. An increase in IL-22 levels prevented inflammation of the GIT [213,214].

The over-activation of AhR, triggered by high levels of certain ligands, can lead to endothelial dysfunction and AS, embryogenesis, neurogenesis, circadian rhythm, aging, metabolism, and hypoxia [215,216,217]. It is, thus, important to study the pathways involved in AhR signaling, including the effect of feedback control by CYP1A1 on AhR signaling. Schiering et al. [218] have shown that the inactivation of *Cyp1a1* in mice suppressed the production of AhR-ligands in the intestinal tract and converted AhR signaling to a pseudo-deficient state. The constitutive expression of *Cyp1a1*, on the other hand, resulted in the loss of AhR-dependent type 3 innate lymphoid cells and T helper 17 cells, which increased susceptibility to enteric infections [218]. The blocking of AhR signaling could, thus, be a new target for the treatment of AS and other CVDs. The exact mechanisms involved in the regulation of AhRs and vascular homeostasis require more research.

## 8. Restriction of Blood Flow

One of the symptoms of AS is the hardening of arteries. Furthermore, macrophages (foam cells) saturated with ox-LDL accumulate on the intima of coronary arteries, the aorta, and the internal carotid artery [219,220,221], resulting in the asymmetric thickening of arteries and the restriction of blood flow [222,223,224]. Blood flow is further restricted by the recruitment of monocytes to inflamed areas with assistance from MCP-1 [225]. The transport of ox-LDL into macrophages is facilitated by lipid rafts, composed of glycosphingolipids, cholesterol, and structurally complex proteins located in the plasma membrane of ECs [115,226]. Although lipid rafts do not directly block blood flow, their disruption by certain drugs such as methyl-beta-cyclodextrin (MβCD), used to control cholesterol levels, may lead to endothelial dysfunction and AS [227]. In antibody-mediated thrombosis, experienced with antiphospholipid syndrome (APS), anti-beta2-glycoprotein I (anti-β2-GPI) reacts with β2-GPI, annexin A2 (ANXA2), TLR2, and TLR4 within lipid rafts. This initiates the release of TNFα and the accumulation of IL-6 [227]. Elevated plasma levels of TNF-α and IL-6 induce the expression of procoagulant proteins such as tissue factor (TF), which leads to coagulation and blood clotting [228]. TLRs and IL-6 negate immune signaling by ECs [229] and the expression of the glycoproteins VCAM-1 and ICAM-1 lectins, such as P-selectin and E-selectin [189]. VCAM-1 binds to alpha 4 beta 1 (α4β1) integrin (very-late-antigen 4, VLA-4) and attracts leucocytes to the surface of ECs [230,231]. Leucocytes are also recruited to ECs by ICAM-1, P-selectin glycoprotein ligand-1 (PSGL-1), and E-selectin [232]. Selectins, present on the surface of ECs, have a strong affinity to sialic-acid-rich epithelial mucin (sialomucins) [233] and mediate interactions between leukocytes, platelets, and ECs, resulting in hemostasis and thrombosis [234,235]. Selectins and the genes encoding selectin (e.g., P-selectin (*SELP*) and P-selectin glycoprotein ligand-1 (*SELPG*) [236]) may be explored as biomarkers for atherosclerosis and other CVDs. Elevated levels of P-selectin were detected in human umbilical vein endothelial cells (HUVECs) of the newborns of parents with a strong family history of myocardial infarction [237].

To protect ECs from further damage, VSMCs migrate from the tunica media to the tunica intima, thickening the arterial wall [222,238,239]. This also leads to the secretion of extracellular matrix (ECM) proteins, such as collagen, elastin, and proteoglycans, which transform fatty streaks (foam cells) into stable plaques, further restricting blood flow [240,241]. Plaque buildup is normally observed in the intima of medium- and large-sized arteries, notably in areas where the endothelial lining of blood vessels is less exposed to hemodynamic shear stress [174] and at sites of inflammation [242]. With an increase in inflammation and the production of ROS, such as superoxide anions (O_2_^•−^) and hydrogen peroxide (H_2_O_2_) produced by NADPH oxidases (NOX), particularly NOX_2_ and NOX_4_ isoforms, plaques become less stable and disrupt, forming blood clots [243,244,245]. Statins, prescribed to lower cholesterol (by inhibiting the enzyme 3-hydroxy-3-methylglutaryl coenzyme A; HMG-CoA), also inhibit NOX and suppress the production of ROS [246]. This may, thus, prevent AS. As time progresses, calcium accumulates in plaques that either partially obstruct or completely block blood flow [247].

An increase in ox-LDL leads to the accumulation of cytokines and other chemokines, such as growth-related oncogene-alpha (GRO-*α*, also known as CXCL1) [248]. GRO-*α* regulates the recruitment of neutrophils and promotes tumor growth, angiogenesis, and metastasis (spreading of cancer cells) [249]. Although not directly involved with blood clotting, GRO-*α* levels rise with an increase in inflammatory reactions following an injury, which may indirectly influence the healing process [250].

Short-term exposure to high levels of ox-LDL and minimally modified LDL (mmLDL) activates the production of transmembrane protein CD16 (FcγRIIIA) on the surface of monocytes, natural killer (NK) cells, neutrophils, and some T cells [251]. An increase in FcγRIIIA stimulates the production of inflammatory cytokines and activates MMPs that contribute to the instability of plaques [252,253]. By exploring mechanisms to inhibit MMP production, we may be able to improve vascular endothelial functions. The inhibition of MMPs may also reduce tumor growth and the spreading of cancer cells (metastasis) [254]. However, dysregulation of MMP may lead to cartilage degradation (arthritis) and the suppression of angiogenesis [255].

## 9. Conclusions

Early signs of AS are usually detected by checking blood pressure and abnormalities in heart rhythm. The latter is detected with an electrocardiogram (ECG or EKG). Calcium buildup in arteries is diagnosed with computed tomography (CT) scans, and arterial blockage is visualized through an angiogram and X-rays. Tissue damage is detected by ultrasound and magnetic resonance imaging (MRI). Final confirmation of AS is usually done by inserting a catheter into the coronary arteries. Other blood flow tests include Doppler ultrasonography and multigated acquisition radionuclide angiography (MUGA). Areas not receiving blood are identified with a thallium/myocardial perfusion scan (nuclear stress test). Although these tests are critically important to detect AS and CVDs, we lack tests to detect early signs of AS. Since butyrate regulates AS, it makes sense to focus on receptors, such as PPAR; TLR; AhR; butyrate-influenced signal-generating pathways, such as MAPK and NLRP3 inflammasome; and ox-LDL transporters. Furthermore, by studying changes in immune reactions, we may identify unique signaling molecules to detect AS much earlier. In-depth studies on the interactions between butyrate and receptors on IECs and ECs, specifically GPCRs (FFARs), provide a solid basis for the intervention of AS and may lead to the design of novel GPCR-targeted drugs. Modulating the chemical structure of GPCRs may increase their sensitivity to GPCR-targeted drugs. Most studies on the influence of butyrate on AS were performed in vitro and in vivo on animals. The influence of butyrate and butyrate-producing gut bacteria on AS must be confirmed by large-scale clinical trials.
